# The Role of Enriched Microbial Consortium on Iron-Reducing Bioaugmentation in Sediments

**DOI:** 10.3389/fmicb.2017.00462

**Published:** 2017-03-20

**Authors:** Yuanyuan Pan, Xunan Yang, Meiying Xu, Guoping Sun

**Affiliations:** ^1^Guangdong Provincial Key Laboratory of Microbial Culture Collection and Application, Guangdong Institute of MicrobiologyGuangzhou, China; ^2^School of Bioscience and Bioengineering, South China University of TechnologyGuangzhou, China; ^3^State Key Laboratory of Applied Microbiology Southern ChinaGuangzhou, China; ^4^Guangdong Open Laboratory of Applied MicrobiologyGuangzhou, China

**Keywords:** iron reducing bacteria, bioaugmentation, consortium, river sediments, high-throughput sequencing, microbial response

## Abstract

Microbial iron reduction is an important biogeochemical process and involved in various engineered processes, including the traditional clay dyeing processes. Bioaugmentation with iron reducing bacteria (IRB) is generally considered as an effective method to enhance the activity of iron reduction. However, limited information is available about the role of IRB on bioaugmentation. To reveal the roles of introduced IRB on bioaugmentation, an IRB consortium enriched with ferric citrate was inoculated into three Fe(II)-poor sediments which served as the pigments for Gambiered Guangdong silk dyeing. After bioaugmentation, the dyeabilities of all sediments met the demands of Gambiered Guangdong silk through increasing the concentration of key agent [precipitated Fe(II)] by 35, 27, and 61%, respectively. The microbial community analysis revealed that it was the minor species but not the dominant ones in the IRB consortium that promoted the activity of iron reduction. Meanwhile, some indigenous bacteria with the potential of iron reduction, such as *Clostridium*, *Anaeromyxobacter*, *Bacillus*, *Pseudomonas*, *Geothrix*, and *Acinetobacter*, were also stimulated to form mutualistic interaction with introduced consortium. Interestingly, the same initial IRB consortium led to the different community successions among the three sediments and there was even no common genus increasing or decreasing synchronously among the potential IRB of all bioaugmented sediments. The Mantel and canonical correspondence analysis showed that different physiochemical properties of sediments influenced the microbial community structures. This study not only provides a novel bioremediation method for obtaining usable sediments for dyeing Gambiered Guangdong silk, but also contributes to understanding the microbial response to IRB bioaugmentation.

## Introduction

Microbial iron reduction as a fundamental biogeochemical process widely exists in the freshwater sediments. It is regarded as the crucial mediator in the carbon, nitrogen, sulfur, and phosphorus cycles (Li et al., 2012). Furthermore, iron reduction plays an important role on degradation of organic contaminants (Zhang et al., 2013; Baek et al., 2016) and bioremediation of toxic metal compounds (Hassan et al., 2015; Si et al., 2015), and particularly, it is also vital for sediments used in environmental-friendly and traditional clay dyeing processes such as mud-tannic dyeing techniques (Pan et al., 2016). Therefore, investigating iron reduction is crucial to understand biogeochemical dynamics in natural sediment environments.

Microbial iron reduction in the sediments depends on quantity and activity of iron reducing bacteria (IRB) and the amounts of reducible Fe(III) such as amorphous Fe(III) oxides (Lovley, 2006). Therefore, in order to increase the activity of iron reduction, bioaugmentation could be an effective, economical and environmental-friendly approach to provide sufficient microbes with special functions. Since the pure specialized strains often failed to compete with indigenous bacteria in the sediment environments (Thompson et al., 2005), consortia with higher diversity were considered as a better choice to enhance the activity of iron reduction. Although a few studies have reported the possibility and efficiency of bioaugmentation with IRB to enhance iron reduction and then affect the performance of anaerobic digestion (Baek et al., 2016), the roles of microbial communities behind the effects remain unknown, such as the survival of introduced consortium and the shift of microbial communities. There are two reasons as follows. Firstly, due to insufficient sequences, the traditional molecular techniques such as denaturing gradient gel electrophoresis and terminal restriction fragment length polymorphism could not exactly monitor the change of microbial communities (Gao and Tao, 2012; Baek et al., 2016). Secondly, the low resolution of traditional techniques leads to difficulty tracking the colonization and succession of the introduced microorganisms (Lentini et al., 2012), especially for IRB consortium with no specific functional gene marker. Nowadays, as the developments of the next generation sequencing, high-throughput microbial community analysis has been applied to study the dynamic succession of environmental microbial communities (Zhou et al., 2014; Zhao et al., 2016). With this method, researchers could track the abundance of introduced consortium without any specific gene marker, and link the succession of microbial communities including indigenous bacteria to the change of physicochemical properties in IRB bioaugmentation system.

Currently, two kinds of viewpoints were proposed about the key factor that influenced the bioaugmentation performance with selected strains or consortia. In general, many studies found that the survival and function of introduced strains were the vital factors influencing the bioaugmentation performance (Herrero and Stuckey, 2015; Baek et al., 2016). In contrast, several studies suggested that there was no direct relation between the abundance of introduced strains and bioaugmentation performance. They speculated that the shift of indigenous microbial community enhanced the performance (Qu et al., 2015; Xun et al., 2015). In terms of the bioaugmentation of IRB consortium, it still needs further investigation to ensure which factor affects the iron respiration activity.

In this study, amending the sediment for Gambiered Guangdong silk was served as a case to study microbial effects during IRB bioaugmentation. Generally, traditional dyeing crafts were obtained through the reaction between the ferric iron clay minerals (e.g., goethite, hematite, palygorskite, and akaganeite) and natural organic dyes, for example, the well-known Maya Blue (Van Olphen, 1966), Bogolan cloth in Mali (Hilu and Hersey, 2005), and Amami Oshima Tsumugi (Wakimoto et al., 2004). Nevertheless, the sediment with abundant precipitated Fe(II) was vital for Gambiered Guangdong silk and it reacted with tannins pre-adsorbed on the silk to form shiny black color (Pan et al., 2016). Usually, the available sediment could be obtained from natural environments. However, with the increasing water pollution, clean sediment is decreasing dramatically. Bioaugmentation with IRB could be a potential technology for exploring new sources of ideal sediment and increasing the recycle and re-use rate of sediment. Unfortunately, very limited information was available on this sustainable method. Therefore, the aim of the present study was to (1) evaluate the feasibility of bioaugmenting three Fe(II)-poor sediments with the enriched IRB consortium; (2) explore microbial response including indigenous and exogenous microbes to IRB bioaugmentation via high-throughput sequencing; and (3) identify the dominant microorganisms which promoted sediment dyeability.

## Materials and Methods

### Materials

Three original river sediments (Orig-SD1, Orig-SD2, and Orig-CH) were collected from creeks in Pearl River Delta, China. Sediments were passed through a 100-mesh standard sieve (0.15 mm opening size) and kept in a cold room (4°C) in darkness for later use. Orig-SD1 and Orig-SD2 were identified as useable sediments because fabrics coated with them met the color demand of Gambiered Guangdong silk (one side: shiny black; the other side: brown; Pan et al., 2016), while Orig-CH was unusable. The physicochemical characteristics and performance details of these three original sediments were listed in Supplementary Table S1. In order to test the bioaugmentation performance, three modified sediments with little ferrous were considered, including high temperature (121°C) and pressure (0.1 MPa) oxidized Orig-SD1 (SD1), air-dried Orig-SD2 (SD2), and unusable Orig-CH (CH).

The silk textiles used in this study were prepared in the ChengYi factory (Foshan, China), which has been dyed with the tannin extract of Ju-liang roots to form a brown color. The prepared textiles (Orig-textile, the color characteristics seen in **Table 1**) were cut into 2 cm × 2 cm square pieces for sediment coating.

**Table 1:** Color characteristics of mud inoculated with or without the enriched IRB consortium.

Sediments	Sediment-coated side			Back side		
		
	L_1_^∗^	a^∗^_1_	b^∗^_1_	L_2_^∗^	a^∗^_2_	b^∗^_2_
SD1W	29.51	12.07	10.33	-	-	-
SD1S	23.11	0.29	0.98	34.36	8.78	13.6
SD2W	26.11	4.62	2.91	-	-	-
SD2S	24.91	1.32	0.95	35.43	11.13	13.17
CHW	28.52	10.73	6.88	-	-	-
CHS	24.86	0.9	1.43	37.15	11.04	14.25
Orig-SD1	24.39	0.32	0.91	36.42	10.86	14.12
Orig-textile	28.17	12.38	9.75	39.90	10.81	15.42


### Enrichment Culture of IRB

Enrichment cultures were prepared in 20-mL serum bottles containing 15 mL ferric citrate medium (g L^-1^: ferric citrate, 3.4; NH_4_Cl, 1.0; KHPO_4_, 0.25; K_2_HPO_4_.3H_2_O, 0.72; CaCl_2_.2H_2_O, 0.07; MgSO_4_.7H_2_O, 0.6; and glucose, 10) and 1 g Orig-SD1 as the inoculants which were sealed with Teflon-coated butyl rubber septa and aluminum crimp caps (Wang et al., 2008). All culture bottles were incubated at 30°C in the dark. The consortium was subcultured in a new serum bottle with 15 mL fresh culture medium once the enrichment cultures turned light green or colorless from yellow color (2–7 days, given by ferric citrate redox indicator). The subculture was consecutively repeated five times. The IRB culture was washed twice and then suspended with sterilized deionized water (OD600 = 1.0) before it was used as bioaugmentation inoculants.

### Bioaugmentation Process

The bioaugmentation experiment procedures are follows: an aliquot (3 g) of the unusable sediments (SD1, SD2, and CH) transferred into the 10-mL vial. Three milliliters of enriched consortium were added into the three sediments, uniformly mixed and cultured at 30°C in the dark for 7 days (SD1S, SD2S, and CHS). Sediments added with sterilized water were used as controls (SD1W, SD2W, and CHW). Each treatment was with three replicates. After 7 days, all sediments with and without bioaugmentation were used to coat the textiles prepared as mentioned above. After reacting for 1 h, the rest sediment on the textile was washed away and the textiles were dried in the sun.

### Characteristics of Inoculated Sediments and Sediment-Coated Textiles

After incubation, a thin layer of sediment was used to coat the Orig-textile for 1 h according to our previous method (Pan et al., 2016) and then the rest sediment was washed away. The color characteristics of both sides of sediment-coated textiles were evaluated with the Commission International d’Eclairage (CIE) Lab coordinates by USPRO Colorimeter (Datacolor 110TM, USA). Optical source was D65, viewing angle 10° and measure diameter 10 mm. The CIELAB color system is organized with three axes in a spherical form: L^∗^, a^∗^, and b^∗^. L^∗^ is associated with the lightness of the color and moves from top (100, white) to bottom (0, black), whereas a^∗^ and b^∗^ are associated with changes in redness–greenness (positive a^∗^ is red and negative a^∗^ is green) and in yellowness–blueness (positive b^∗^ is yellow and negative b^∗^ is blue). The L^∗^, a^∗^, and b^∗^ were calculated from three repetitive measurements for every sample. And color difference (Δ*E*) between textile samples was used as the indicator on judging the dyeability of sediments and calculated according to the Eq. 1. From the point of technical dyeing using natural raw dyes, a somewhat wider color difference of Δ*E* ≤ 3 could be permitted with the use of natural resources (Martínez et al., 2001).

$$\Delta E=\sqrt{{\left(L_{i}^{*}-L_{j}^{*}\right)}^{2}+{\left(a_{i}^{*}-a_{j}^{*}\right)}^{2}+{\left(b_{i}^{*}-b_{j}^{*}\right)}^{2}}$$

*L_i_^∗^, a_i_^∗^, b_i_^∗^*: color values of the *i* textile;

*L_j_^∗^, a_j_^∗^, b_j_*^∗^: color values of the *j* textile.

The sediment pH and oxidation-reduction potential (ORP) were monitored with a S20 K pH meter (Mettler Toledo, Switzerland). The organic matter content was estimated according to the previous method (Haller et al., 2011) except extended to 4 h. HCl-extractable Fe(II) and total Fe (TFe) in the sediment were extracted with 0.5 mol/L HCl for 1 h (Lovley and Phillips, 1987). After centrifuged at 8000 rpm for 5 min, the supernatant was determined using the 1,10-phenanthroline colorimetric method at 510 nm on a full wavelength scanner (Thermo Scientific, MULTISKAN GO). TFe including Fe(III) and Fe(II) in the sediment was extracted with hydroxylamine hydrochloride and determined as the Fe(II) determination (Lovley and Phillips, 1987).

### DNA Extraction and Sequencing

DNA was extracted from 250 mg of sediment samples using the PowerSoil^TM^ DNA Isolation Kit (Mo Bio Laboratories, Carlsbad, CA, USA) according to the manufacturer’s instructions. The bacterial 16S rRNA genes were amplified using the PCR primers 515f/806r targeting the V4 region (Pylro et al., 2014). To distinguish the different samples, a Barcoded-tag with six nucleotide bases was randomly added to the upstream of the universal primer. The primers which were added with Barcoded-tag sequences were Barcoded-tag fusion primers. After quantification and quality control, PCR products were gradually diluted and quantified. The V4 tag PCR products were pooled with the other samples and sequenced using 300 bp paired-end model with the Illumina MiSeq platform at Chengdu Institute of Biology (Chengdu, China).

### Sequence Data Analysis

After sequencing, the final V4 tag sequences were assembled through finding the overlap between paired-end reads by the FLASH software. Chimeras were identified via UCHIME algorithm on mothur platform. Low quality fragments were filtered out using QIIME software. Sequences were clustered to operational taxonomic units (OTUs) at 97% sequence similarity by using UCLUST software (Edgar, 2010). Singletons were removed from the whole sequence data set and each sample was randomly sampled and normalized at 11,000 sequences. The numbers of original reads and final OTUs are listed in Supplementary Table S2. The dissimilarity test [non-metric multidimensional scaling (NMDS)] based on Bray–Curtis similarity distance matrices were performed by the Vegan package in R 3.1.3. The dominant OTUs in each group were depicted in a heat map conducted with R 3.1.3, and canonical correspondence analysis (CCA) was used to analyze the relationship between these OTUs and sediment properties with Mantel test. The Illumina sequence raw data reported here was submitted to the NCBI Sequence Read Archive (http://www.ncbi.nlm.nih.gov/sra) under accession number SRP083001.

## Results

### Performance of Bioaugmented Sediments

After 7-day incubation, all of the three bioaugmented sediments (SD1S, SD2S, and CHS) revealed higher dyeabilities than their control groups (**Table 1**). The bioaugmented sediments performed shiny black color on the sediment-coated side (*L*^∗^_SD1S_ = 23.11; *L*^∗^_SD2S_ = 24.91; *L*^∗^_CHS_ = 24.86), comparable with the standard cloth dyed with Orig-SD1 (Δ*E* < 3). Furthermore, the bioaugmented sediments did not penetrate to the back and the backside still kept brown (Δ*E*_SD1S_ = 2.97; Δ*E*_SD2S_ = 1.41; Δ*E*_CHS_ = 0.84), which met the demand of two tone colors for Gambiered Guangdong silk. In contrast, the control sediments (SD1W, SD2W, and CHW) still kept the original brown color (*L*^∗^_SD1W_ = 29.51; *L*^∗^_SD2W_ = 26.11; *L*^∗^_CHW_ = 28.52), near to the color of Orig-textile (*L*^∗^_Orig-textile_ = 28.17).

### Sediment Properties

In order to clarify the influences of IRB inoculation on sediment dyeability, characteristics of sediments were investigated (**Table 2**). The pH and LOI of the bioaugmented groups showed a slight increase and decrease, respectively, compared to the control group, except for the pH of SD1S. As expected, the concentration of HCl-extractable Fe(II) in bioaugmented sediments were significantly higher than those in controls (*p* < 0.01), increasing by 3.5-, 12-, and 83-fold, while there was no significant change in HCl-extractable TFe (**Table 2**). In addition, the ORP results were well consistent with the Fe(II) results: the controls had positive ORP with oxidizing conditions while the IRB bioaugmented groups had negative ORP with reducing conditions for iron reducing.

**Table 2:** Physiochemical characteristics and dyeability evaluation of sediments inoculated with or without iron reducing consortium.

Sediment	Total Fe (μmol g^-1^)^a^	Fe(II) (μmol g^-1^)^b^	pH	ORP	LOI (%)	Δ*E*_1_/Δ*E*_2_
SD1W	224.38 ± 14.97	25.56 ± 17.22	6.69 ± 0.03	194.6 ± 15.70	7.29 ± 1.26	15.91/–
SD1S	247.05 ± 26.13	114.35 ± 34.24^∗∗^	6.63 ± 0.06	-102.13 ± 48.31^∗∗^	6.54 ± 0.48	1.29/2.97
SD2W	234.24 ± 45.36	12.21 ± 0.36	6.98 ± 0.21	20.68 ± 1.03	8.08 ± 0.84	5.06/–
SD2S	271.46 ± 11.02	86.69 ± 6.66^∗∗^	7.08 ± 0.11	-64.28 ± 18.44^∗∗^	7.51 ± 0.36	1.14/1.41
CHW	140.14 ± 6.92	0 ± 3.52	5.09 ± 0.07	243.13 ± 28.81	9.23 ± 0.62	12.70/–
CHS	136.91 ± 23.41	83.98 ± 16.46^∗∗^	5.61 ± 0.14^∗∗^	-56.05 ± 25.51^∗∗^	8.11 ± 0.67	1.02/0.84


*LOI, organic matter; ORP, redox potential; total Fe, 0.5 mol L^-1^ hydroxylamine hydrochloride extraction; Fe(II), 0.5 mol L^-1^ HCl extraction; Δ*E*_1_ and Δ*E*_2_ represents the color difference of sediment-coated side and back side compared with Orig-SD1, respectively. Δ*E* < 3 indicated the good dyeability. ^∗∗^*p* < 0.01 in *t*-test.*

### Microbial Community Analysis in Enriched IRB Consortium and Sediments

**FIGURE 1:**
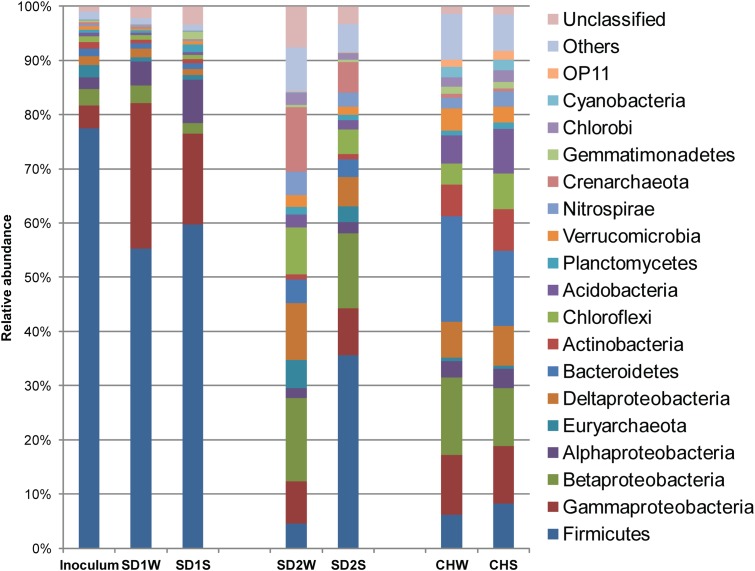
**Diversity in the bacteria communities at the phylum level in response to iron reducing consortium treatments for the three sediments, identified by 16S rRNA gene sequences.** The bacterial phyla represented by >1% of total sequences are presented here. Phylum making up less than 1% of the total sequences were classified as others.

Eleven thousand effective sequences were re-sampled from each sediment sample, resulting in 1,440–3,759 OTUs at 97% sequence identify cutoff (Supplementary Table S2) and being assigned to different taxa (**Figure 1**). In the enriched iron reducing consortium, Firmicutes (77.4%) was the overwhelmingly dominant phylum, followed by Proteobacteria with 11.0%. Among the Firmicutes phylum, nearly 44.3% sequences was assigned to genus *Clostridium*. At the OTU level, one-third sequences were assigned to *Clostridium* sp. (denovo 173227).

**FIGURE 2:**
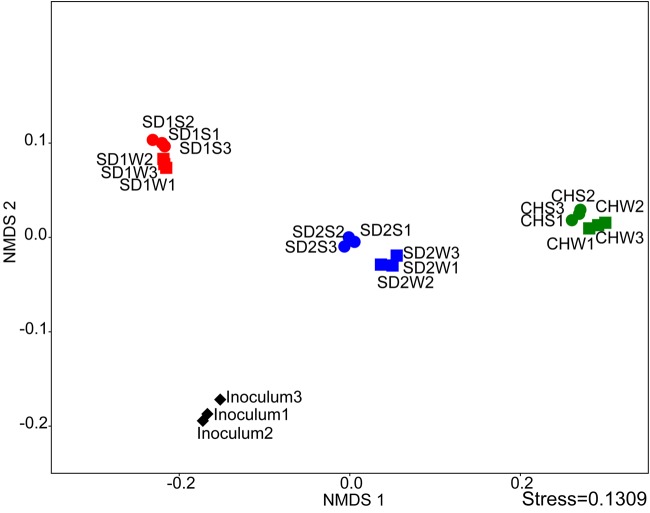
**Comparing the microbial communities between inoculated groups and the controls using NMDS analysis at the genus level**.

The NMDS analysis was applied to analyze sediments microbial communities with and without bioaugmentation. Although the data points of sediments with the same source were adjacent, there was a distinction between bioaugmented and non-bioaugmentation samples. Moreover, the succession of microbial communities proceeded in different directions of community succession (**Figure 2**).

**FIGURE 3:**
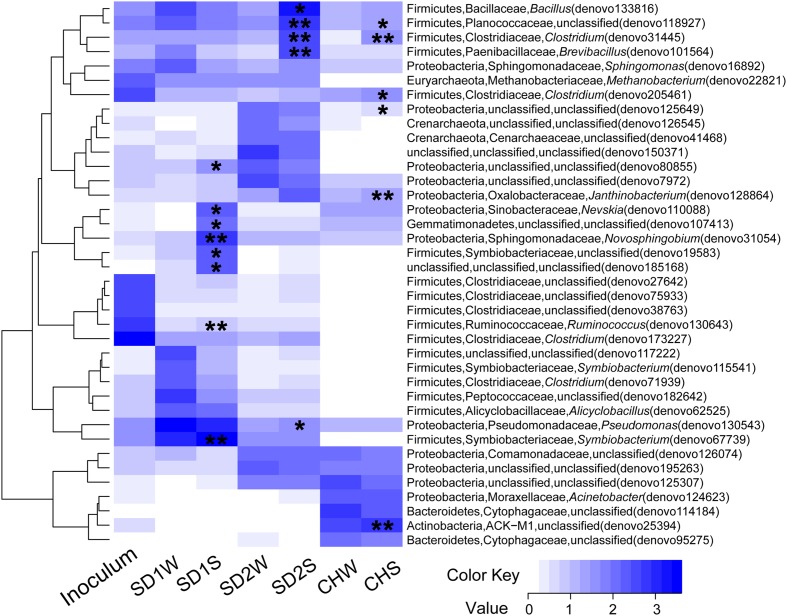
**Heatmap of the enriched iron reducing consortium and sediments with and without inoculation based on the log_10_ (sequence numbers +1) of the 38 OTUs whose reads occupied above 1% of total sequences.** The phylum, family, and genus for each OTU were showed in the figure. ^∗^ and ^∗∗^ indicated this OTU with a significant increase in the inoculated sediments (SD1S, SD2S, and CHS) compared to the relative controls (SD1W, SD2W, and CHW). ^∗^0.01 < *p* < 0.05, ^∗∗^*p* < 0.01.

In details, in terms of the phylum level (**Figure 1**), Firmicutes and Proteobacteria dominated in SD1 and SD2, while the CH community was mainly dispersed by Firmicutes, Proteobacteria, and Bacteroidetes. After bioaugmentation, the average abundance of Firmicutes increased from 55.3% (SD1W), 4.6% (SD2W), and 6.2% (CHW) to 59.7% (SD1S), 35.6% (SD2S), and 8.3% (CHS), respectively, but total percentages of Proteobacteria in all sediments decreased, even by 8.2% for SD1S. At the genus level (Supplementary Figure S1), bioaugmentation with IRB consortium led to a significant increase in the relative abundance of genera *Symbiobacterium* (by 24.0%), *Planctomyces* (by 1.1%), *Geobacter* (by 0.2%), *Novosphingobium* (by 5.8%), and *Nevskia* (by 1.7%) in SD1; *Clostridium* (by 3.0%), *Bacillus* (by 13.9%), *Brevibacillus* (by 2.8%), and *Janthinobacterium* (by 1.6%) in SD2; *Clostridium* (by 0.9%), *Anaeromyxobacter* (by 0.6%), and *Geothrix* (by 0.5%) in CH. In addition, the dominant 38 OTUs (>1% total sequences for each sample) of all the samples were analyzed (**Figure 3**), which were mainly composed of Firmicutes (17 OTUs) and Proteobacteria (12 OTUs). The major species (e.g., denovo 173227) in the IRB consortium showed no dominant position in bioaugmented sediments. The number of the significantly increased OTUs in bioaugmented sediments were 8 (SD1S), 5 (SD2S), and 6 (CHS), respectively, and some of these OTUs were different among the three sediments. Moreover, some sediment-specific (indigenous) IRB increased and be only found in their corresponding sediment (Supplementary Table S3).

### Potential IRB

The relative abundance of potential iron reducers were listed in **Table 3**. The genus *Clostridium* was the most abundant genus in the IRB consortium, but just occupied 1.11, 3.55, and 1.20% in SD1S, SD2S, and CHS, respectively. *Pseudomonas* (11.64%) was the most abundant genus of IRB in SD1S. *Bacillus* (14.19%) and *Brevibacillus* (2.84%) were the predominant iron-reducing genus in SD2S and increased by 50- and 46-fold, respectively, compared with the SD2W. Differently, the iron reducing genera for CHS were dispersed and composed of *Anaeromyxobacter* (1.37%) and *Geothrix* (1.07%) both increased by about twofold. The significantly increased OTUs in the sediments but not in the IRB consortium (Supplementary Table S3), were classified to several genera which were reported to own the ability of iron reduction such as *Anaeromyxobacter* (Breidenbach et al., 2015), *Bacillus* (Alabbas et al., 2013), *Clostridium* (Hassan et al., 2015), *Azospira* (Peng et al., 2016) *Paenibacillus* (Petrie et al., 2003), *Desulfosporosinus* (Bertel et al., 2012), and *Treponema* (Baek et al., 2016).

**Table 3:** The potential iron reducing bacteria in different phylogenetic OTUs taxa obtained by pyrosequencing of 16S rRNA genes using Miseq platforms.

Taxonomic description	Percent of total sequence (%)						
	
	Consortium	SD1W	SD1S	SD2W	SD2S	CHW	CHS
Firmicutes							
Clostridia							
Clostridiaceae							
*Clostridium*	**44.25**	**4.08**	**1.11**	0.53	**3.55**^∗∗^	0.34	**1.20**^∗∗^
Peptococcaceae							
*Desulfosporosinus*	0.12	**2.02**	**0.99**	0.05	0.14	0.02	0.06
Bacilli							
Bacillaceae							
*Bacillus*	0.54	**2.62**	0.61	0.28	**14.19**^∗∗^	0.32	0.34
Paenibacillaceae							
*Brevibacillus*	0.17	**1.46**	0.34	0.06	**2.84**^∗∗^	0.03	0.03
Proteobacteria							
Betaproteobacteria							
Pseudomonadaceae							
*Pseudomonas*	0.44	**23.37**	**11.64**	0.25	**1.31**	0.42	0.40
Deltaproteobacteria							
Myxococcaceae							
*Anaeromyxobacter*	0.13	0.05	0.05	0.29	0.28	0.76	**1.37**^∗∗^
Geobacteraceae							
*Geobacter*	0.17	0.10	0.27^∗∗^	0.45	0.27	0.83	**1.06**
Gammaproteobacteria							
Moraxellaceae							
*Acinetobacter*	0.05	0.02	0.01	0.01	0.02	**3.52**	**3.35**
Acidobacteria							
Holophagae							
Holophagaceae							
*Geothrix*	0.00	0.00	0.00	0.05	0.04	0.54	**1.07**^∗∗^


*Consortium represented for the enriched iron reducing bacteria. ^∗∗^Indicated that the relative abundances in inoculated samples were higher than those in their controls significantly (*p* < 0.01). Bold numbers indicate the most important percent of total sequence. The potential iron reducing genera were classified according to the published references, *Clostridium* (Hassan et al., 2015), *Desulfosporosinus* (Bertel et al., 2012), *Bacillus* (Alabbas et al., 2013), *Brevibacillus* (Ding et al., 2015), *Pseudomonas* (Naganuma et al., 2006), *Anaeromyxobacter* (Breidenbach et al., 2015), *Geobacter* (Lentini et al., 2012), *Acinetobacter* (Hassan et al., 2015), and *Geothrix* (Nevin and Lovley, 2002).*

### Relationship between Microbial Community and Sediment Properties

**FIGURE 4:**
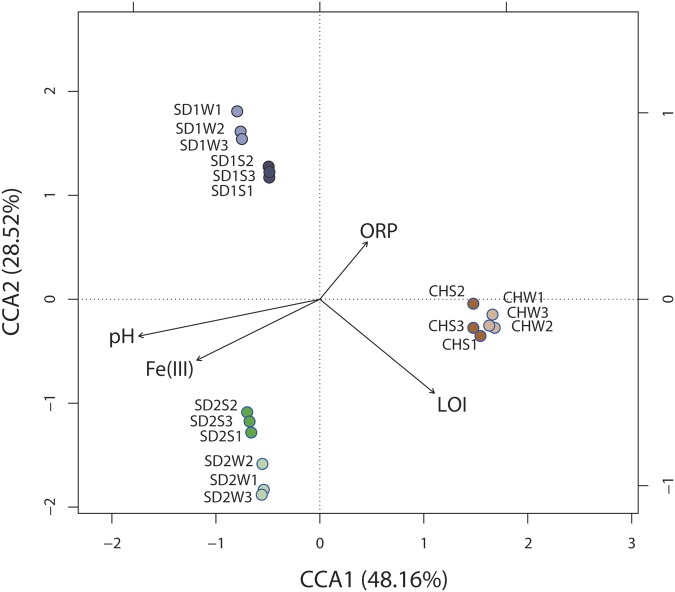
**Canonical correspondence analysis (CCA) of microbial community structures (OTUs) from six sediment samples with respect to the four environmental variables.** Arrows indicate the direction and magnitude of measurable variables associated with community structures.

Significant correlation was observed between the composition of the bacterial community (OTU level) and physiochemical properties [pH, ORP, Fe(III), LOI] of sediments with different sources through the Mantel test (*p* < 0.01) and canonical correspondence analysis (CCA) (**Figure 4**). CCA showed the first two components (CCA1 and CCA2) together explained 76.68% of the total variation of sediment microbial community. Although some parameters [e.g., Fe(II) and ORP] had significant changes after bioaugmentation (**Table 2**), greater sediment-to-sediment difference than treatment-to-control in CCA profile (**Figure 4**) implied that the whole physiochemical properties were the substantive influence on the bacterial community constitution.

## Discussion

While microbial iron reduction in the sediment environments has been concerned for several decades, little has been done to characterize bioaugmented iron reduction with IRB consortium. In this study, we successfully enhanced iron reduction and then achieved the goal of improving the sediment dyeability for Gambiered Guangdong Silk by inoculating enriched IRB consortium (**Table 1**). As expected, the concentrations of HCl-extractable ferrous in all the bioaugmented sediments increased by 35% (SD1), 26% (SD2), and 61% (CH) comparing with the control groups, respectively (**Table 2**). Meanwhile, the negative ORP with reducing conditions favored the precipitation of ferrous (Bongoua-Devisme et al., 2013). These results were coincident with our previous study which suggested that precipitated ferrous iron in the sediment was the key factor for the success of dyeing technique of Gambiered Guangdong silk (Pan et al., 2016).

To explain that the enhanced iron reduction was ascribed to either the survival of introduced IRB consortium or the shift of indigenous microorganisms, the microbial communities were taken insight through high-throughput technique. In general, the dominant bacteria would be responsible for the enhanced performance. Unexpectedly, the dominant specie (denovo 173227) in enriched IRB consortium, which accounted for 42.95% of total sequences and assigned to potential iron-reducing genus *Clostridium* (Li et al., 2011; Peng et al., 2016), did not proliferate significantly in bioaugmented sediments and only accounted for little percentages (0.22, 0.21, and 0%, respectively, in SD1S, SD2S, and CHS). Moreover, the other major OTUs in the consortium also lost their dominant position after augmented into sediments (**Figure 3**), which implied the uncertain risk in using the well-growing strains as the augmentation agent. In this study, however, the bioaugmentation was success, which might be attributed to the minor species in the inoculums. Minor species are considered as the seed bank in a microbial community (Pedrós-Alió, 2006; Campbell et al., 2011) and serve as the keystone within complex consortia with the potential to become dominant in response to shifts in environmental conditions (Sogin et al., 2006). These minor species survived in new environment and were responsible for iron reducing function (**Figure 3** and **Tables 2**, **3**). As **Figure 3** illustrated, the minor OTUs once in consortium substantially showed higher abundances in bioaugmented sediments than those in relative controls, and most of them were assigned to several potential iron-reducing genera, such as *Bacillus*, *Brevibacillus*, *Clostridium*, and *Pseudomonas* (**Table 3**). In addition, some indigenous OTUs (only observed in sediments) also increased after bioaugmentation (Supplementary Table S3), which might be attributed to the mutualism that the exogenous species collaborate with the indigenous IRB and perform higher exploitability of Fe(III). These stimulated bacteria and survival species in the consortium formed a multispecies interactive network. Therefore, iron reduction could be functioned not only through proliferating the exogenous minor taxa but also collaborators with the indigenous IRB.

Interestingly, as the **Table 3** and **Figures 2**, **3** shown, the same consortium had triggered the succession in different directions. These might be attributed to the differences in the sediment characteristics (Böer et al., 2009). Distinct OTUs had a differential niche adaptation and tended to adapt to changes in their environments (Storey et al., 2015). Some members might extinct due to poor ability to adapt to changing environments, while others could proliferate in the new environments (Faust and Raes, 2012). Eventually, the relative balanced state of community structure was formed based on the physicochemical characteristics (Zhou et al., 2014; Qu et al., 2015; Sanders, 2016). In the present study, significant correlation was definitely observed between the composition of the bacterial community and such properties of sediments with different sources through the Mantel test (*p* < 0.01) and CCA (**Figure 4**). Furthermore, the bacterial community structure analysis showed reproducible inter-groups and significantly different intra-groups (**Figures 2**, **4**). The results indicated that the environment conditions influenced the microbial communities, which also explained the reason that specific IRB played the roles in different sediments (**Table 3**). That is, because of the allopatric speciation, the same consortium formed different functional assemblies. This implied that inoculating a consortium was equivalent to providing a function library (seed bank); the key contributors, who might used to be minor species, would function in compatible environment. Therefore, it is also suggested that bioaugmentation with microbial consortia might be a better choice than with specialized strains, due to their adaptation to a wider environmental conditions as well as their synergistic interactions.

## Conclusion

The findings demonstrated that sediments bioaugmented with enriched IRB consortium obtained good dyeability for Gambiered Guangdong silk due to the increased Fe(II) concentration. The bioaugmentation process facilitated iron reduction through the mutualism interaction between survived minor species from the IRB consortium and some stimulated indigenous bacteria including *Clostridium*, *Anaeromyxobacter*, *Bacillus*, *Pseudomonas*, *Geothrix*, and *Acinetobacter*. Meanwhile, due to the different physiochemical properties, the same IRB consortium led to the community successions on different directions in the three sediments. This study suggested that consortium might be a better choice than pure strains because of a lower requirement for environmental conditions.

## Author Contributions

YP designed the study, performed experiments, analyzed the data, and wrote the manuscript; XY analyzed the data, interpreted the results, and revised the manuscript; MX and GS revised the manuscript and approved the final version.

## Conflict of Interest Statement

The authors declare that the research was conducted in the absence of any commercial or financial relationships that could be construed as a potential conflict of interest.
