# Supplementary material for: The Role of Enriched Microbial Consortium on Iron-Reducing Bioaugmentation in Sediments
Source: Front Microbiol. 2017 Mar 20;8:462. doi: 10.3389/fmicb.2017.00462 (PMC5357831; doi:10.3389/fmicb.2017.00462)
Supplement: Supplementary file 1 [file Data_Sheet_1.DOCX]

***Supplement of***

**The role of enriched microbial consortium on iron-reducing bioaugmentation in sediments**

Yuanyuan Pan^1, 2, 3, 4^, Xunan Yang^1, 3, 4*^, Meiying Xu^1, 3, 4^, Guoping Sun^1, 3^^, 4*^

1. Guangdong Provincial Key Laboratory of Microbial Culture Collection and Application, Guangdong Institute of Microbiology, Guangzhou, 510070, China

2. School of Bioscience and Bioengineering, South China University of Technology, Guangzhou, 510006, China

3. State Key Laboratory of Applied Microbiology Southern China, Guangzhou, 510070, China

4. Guangdong Open Laboratory of Applied Microbiology, Guangzhou, 510070, China

^*^ Corresponding author:

*E-mail address*: sgpgim@163.com (Guoping Sun) AND yangxn@gdim.cn (Xunan Yang), Guangdong Institute of Microbiology, Guangzhou 510070, China.

Tel.: +86 20 87684471; fax: +86 20 87684471.

**Table S1** Physiochemical characteristics and dyeability evaluation of the originally collected sediments.

| Sediment | pH | ORP | LOI (%) | Total Fe (μmol g^-1^) | Fe(II) (μmol g^-1^) | *△E_1_/△E_2_* | Dyeability |
| --- | --- | --- | --- | --- | --- | --- | --- |
| Orig-SD1 | 7.03 | -221.9 | 7.00±0.16 | 474.87±63.11 | 357.55±21.29 | 0.00/0.00 | Yes |
| Orig-SD2 | 7.20 | -61.7 | 7.40±1.44 | 390.55±11.20 | 359.75±5.29 | 1.45/2.73 | Yes |
| Orig-CH | 4.69 | 371.6 | 8.38±0.05 | 131.99±5.03 | 0±5.14 | 13.11/-- | No |

*Notes:* Orig-SD1 and Orig-SD2 were collected from Lunjiao (Foshan city); Orig-CH was collected from Conghua (Guangzhou city). LOI: organic matter; ORP: redox potential; Total Fe: 0.5 mol L^-1^ hydroxylamine hydrochloride extraction; Fe(II): 0.5 mol L^-1^ HCl extraction; Dyeability: Yes, sediment could be used for dyeing GGS; No, sediment could not be used for dyeing GGS. *△E_1_* (sediment-coated side) and *△E_2_* (back side) represented the color difference of sediment dyed textiles, compared with Orig-SD1.

**Table S2** The numbers of original sequences and OTUs.

| Sample ID | Quality reads | Remove singletons | Remove singletons and resample to 11000 | |
| --- | --- | --- | --- | --- |
|  | Original Seqs^a^ | Treated Seqs | Resampled Seqs | Final OTUs |
| Inoculum1 | 58325 | 54573 | 11000 | 1674 |
| Inoculum2 | 46779 | 43745 | 11000 | 1588 |
| Inoculum3 | 36598 | 34112 | 11000 | 1956 |
| SD1W1 | 71550 | 67399 | 11000 | 1684 |
| SD1W2 | 86370 | 81072 | 11000 | 1706 |
| SD1W3 | 46872 | 44002 | 11000 | 1697 |
| SD1S1 | 40689 | 38058 | 11000 | 1698 |
| SD1S2 | 58029 | 55034 | 11000 | 1440 |
| SD1S3 | 89900 | 84352 | 11000 | 1677 |
| SD2W1 | 62196 | 56215 | 11000 | 3223 |
| SD2W2 | 29690 | 25884 | 11000 | 3388 |
| SD2W3 | 131060 | 121885 | 11000 | 3079 |
| SD2S1 | 413370 | 392610 | 11000 | 2837 |
| SD2S2 | 74607 | 67750 | 11000 | 2800 |
| SD2S3 | 71910 | 65278 | 11000 | 2882 |
| CHW1 | 36535 | 30239 | 11000 | 3448 |
| CHW2 | 18316 | 14568 | 11000 | 3405 |
| CHW3 | 22751 | 18465 | 11000 | 3361 |
| CHS1 | 24042 | 19584 | 11000 | 3604 |
| CHS2 | 21602 | 17131 | 11000 | 3759 |
| CHS3 | 23123 | 18432 | 11000 | 3576 |

Notes: ^a^ Seqs means sequences.

**Table S3** The increased OTUs with potential iron reduction in the sediments but not in the IRB consortium.

| Genus | SD1 (436)^a^ | SD2 (162) | CH (855) |
| --- | --- | --- | --- |
| *Anaeromyxobacter* | -- | -- | denovo203610  denovo130037 |
| *Azospira* | -- | -- | denovo12562 |
| *Bacillus* | -- | denovo104373 ^b^  denovo101954 denovo64704  denovo204083 | -- |
| *Clostridium* | -- | denovo52253  denovo48039 | denovo90017  denovo157486  denovo197344 |
| *Desulfosporosinus* | denovo76872 | -- | -- |
| *Paenibacillus* | denovo205009 | denovo20865 | -- |
| *Treponema* | -- | -- | denovo90262 |

Notes: ^a^ the sequence number of total increased OTUs which were not in the IRB consortium; ^b^ the serial number of OTUs

**Fig. S1** Diversity in the bacteria communities at the genus level in response to iron reducing consortium treatments for the three sediments, identified by 16S rRNA gene sequences. The bacterial genera represented by > 1% of total sequences are presented here.
